# Deficiency of PTEN Confers Hypersensitivity to Fatty Acid-Mediated ER Stress in Transformed Hepatocytes

**DOI:** 10.3390/ijms27062778

**Published:** 2026-03-19

**Authors:** Olaya Yassin, Odai Darawshi, Fangfang Wang, Youwei Zhang, Ata Abbas, William C. Merrick, William Cheung, Antony Antoniou, Shakti P. Pattanayak, Boaz Tirosh

**Affiliations:** 1 Institute for Drug Research, The Hebrew University of Jerusalem, Jerusalem 9112001, Israel; oxy27@case.edu (O.Y.); odai.darawshi@mail.huji.ac.il (O.D.); 2 Department of Biochemistry, Case Western Reserve University School of Medicine, Cleveland, OH 44106, USA; axa335@case.edu (A.A.); wcm2@case.edu (W.C.M.); sxp1286@case.edu (S.P.P.); 3 Department of Pharmacology, Case Western Reserve University School of Medicine, Cleveland, OH 44106, USA; fxw188@case.edu (F.W.); yxz169@case.edu (Y.Z.); 4 Department of Applied Sciences, Northumbria University, Newcastle upon Tyne NE1 8ST, UK; william.cheung@northumbria.ac.uk (W.C.); antony.antoniou@northumbria.ac.uk (A.A.)

**Keywords:** liver cancer, PTEN, ER stress, terminal UPR, free fatty acids, apoptosis

## Abstract

Deletion of the tumor suppressor gene phosphatase and tensin homolog (PTEN) in hepatocellular carcinoma (HCC) is associated with a poor response to therapy and reduced survival. In mice, the deletion of PTEN in hepatocytes generates steatosis; however, on the background of steatosis not all emerging HCC cells lack PTEN, suggesting that steatosis confers a metabolic liability to proliferating PTEN-deficient hepatocytes. Here, we show that PTEN-deficient HepG2 cells develop terminal stress in the endoplasmic reticulum (ER) and profound apoptosis when exposed to a mixture of oleic and palmitic acids, while control cells do not. Lipidomic analyses before and after the treatment indicate a higher increase in triglycerides in PTEN KO cells, as well as profound differences in phospholipid concentrations. Although the triglyceride content increases, the coalescence into lipid droplets was impaired in the KO cells, together with a reduction in β-oxidation. Xenograft studies showed that PTEN KO HCC tumors progressed faster than did the control tumors when mice were fed with normal chow and slower under a high-fat diet. We suggest that while the health risks of a fatty acid-rich diet to liver function and the increased propensity to develop HCC are prominent, once a PTEN-deficient HCC has been established, it exposes vulnerability to lipid overload that can be exploited through diet and pharmacological interventions.

## 1. Introduction

Hepatocellular carcinoma (HCC) is the most common type of liver cancer, originating in hepatocytes. HCC is associated with chronic inflammation and fibrosis arising from different etiologies, viral hepatitis, alcoholism and obesity [1,2,3]. HCC is treated with multikinase inhibitors, the most common being lenvatinib, sorafenib and regorafenib [4,5]. However, on average, patients with advanced HCC only experience ~3 months of benefit from this treatment [6]. The combination of immune checkpoint inhibitors with anti-angiogenic therapy has shown improved survival rates and disease control, adding, on average, an additional 6 months to overall survival [7]. Still, due to late diagnosis and ineffective therapy, HCC is a leading cause of cancer-related death globally [8]. Hence, novel therapeutic and disease management plans are urgently needed to improve the life span and life quality of HCC patients.

The phosphatase and tensin homolog (PTEN) is a phosphoprotein/phospholipid dual-specificity phosphatase that antagonizes the activity of phosphoinositide 3-kinases (PI3K). Deletion of the *PTEN* gene is one of the most frequent mutations in cancer [9]. PTEN converts phosphatidylinositol (3,4,5)-trisphosphate (PIP3) into phosphatidylinositol (4,5)-bisphosphate (PIP2). When PTEN activity is reduced, PIP3 accumulates, acting as an oncogenic second messenger, primarily by activating the AKT/mTOR pathway [10]. As such, PTEN acts as a tumor suppressor. Almost half of HCC tumors exhibit a heterozygote deletion in *PTEN* or reduced expression. This is observed specifically in the tumor cells, while the normal liver parenchyma expresses normal PTEN levels [11,12]. In mice, the hepatocyte-specific deletion of PTEN is sufficient to cause liver cancer within 12–15 months [13]. Prior to HCC development, PTEN deficiency causes liver steatosis. The steatotic phenotype is attributed primarily to the overactivation of the mechanistic target of rapamycin (mTOR), a kinase which promotes many metabolic activities associated with the biogenesis of lipids [14]. The combination of PTEN deletion with additional oncogenic aberrations in HCC, such as the overexpression of c-MET [15], or β-catenin [16], accelerates carcinogenesis. Importantly, in humans, steatotic HCC is often seen in association with high BMI and metabolic diseases such as diabetes or hyperlipidemia and is correlated with a worse prognosis [17]. Accordingly, diet recommendations for HCC patients include an increased intake of fruits, vegetables and whole grains, increased consumption of polyunsaturated, particularly omega-3, fatty acids, and avoiding the consumption of saturated fatty acids as much as possible [18].

Secreted proteins fold and mature in the endoplasmic reticulum (ER). Upon increased demand for secretory capacity, and/or impaired folding, unfolded proteins accumulate in the ER, imposing conditions of ER stress. ER stress is met by adaptive responses to avoid protein aggregation and cell death, in a manner dependent on the duration and intensity of the stress. The main mechanism of adaptation to ER stress in eukaryotic cells is the unfolded protein response (UPR) [19]. In mammalian cells, the UPR comprises three major ER transducers that respond to perturbed ER homeostasis. PERK and ATF6 are unique to mammals, while IRE1α is shared by all eukaryotes, including yeast. In response to ER stress, IRE1α undergoes autophosphorylation, which activates its nuclease activity towards the mRNA of XBP1. This generates the spliced XBP1 (sXBP1) protein, a potent transcription factor that modulates multiple functions in the secretory pathway [20] and is the main driver of gene expression response to ER stress [21]. PERK is activated in a similar manner to IRE1α. Upon autophosphorylation, it phosphorylates eIF2α, leading to a global attenuation in translation and selective translation of specific mRNA molecules, such as of ATF4. Feedback mechanisms driven by ATF4 are then engaged to restore translation [22]. ATF6 is activated through a different mechanism which involves trafficking to the Golgi and intramembrane cleavage, liberating its N-terminus for transcription activity. The mechanisms of activation of UPR transducers are diverse and include a direct binding to unfolded proteins in the ER [23], i.e., a release from interaction with the BiP chaperone [24] and in response to changes in the lipid composition of ER membranes [25,26,27]. The mechanisms by which lipid homeostasis activates the UPR are poorly understood. The UPR is engaged to alleviate ER stress conditions, acquiring pro-survival roles. However, if stress persists, the downstream effectors of the UPR, such as ATF4 and CHOP, promote cell death via various mechanisms [28], a response referred to as the terminal UPR [29].

HCC is characterized by an increased expression of UPR genes, particularly those mediated by PERK [30] and IRE1α [31]. The UPR is also involved in the premalignant stages of HCC, promoting the transition from the asymptomatic fatty liver condition, metabolic dysfunction-associated fatty liver disease (MAFLD), to the inflammatory condition, metabolic dysfunction-associated steatohepatitis (MASH) [32,33]. The underlying reasons for the chronic ER stress in the liver cells are a consequence of lipid dysregulation, such as the accumulation of free cholesterol [34], and a reduction in the ratio between phosphatidylcholine and phosphatidylethanolamine [35]. Mouse models in which chronic ER stress is induced in liver cells in conjunction with *PTEN* deletion show an accelerated HCC initiation and progression [36]. However, a detailed analysis of lipid metabolism in PTEN-deficient HCC and the connection to ER stress has not been reported. Furthermore, how this triad (PTEN/lipids/ER stress) is affected by exogenously provided lipids has not been documented, a gap we address here. We hypothesize that when PTEN expression is compromised in HCC, lipid metabolism is affected in a manner that can be exploited to further enhance ER stress conditions and generate a terminal UPR, specifically in the PTEN KO tumor cells. In the current study, we show that PTEN KO HepG2 cells develop terminal ER stress conditions in the presence of high concentrations of a mixture of oleic acid (OA) and palmitic acid (PA), while control cells do not. We provide evidence that the hypersensitivity to lipid-mediated ER stress conferred by the deficiency of PTEN can be used to selectively compromise the tumorigenicity of PTEN KO liver cancer.

## 2. Results

### 2.1. Deficiency of PTEN in HepG2 Cells Generates Hyperactivation of the UPR in Response to a Mixture of OA and PA

**Figure 1:**
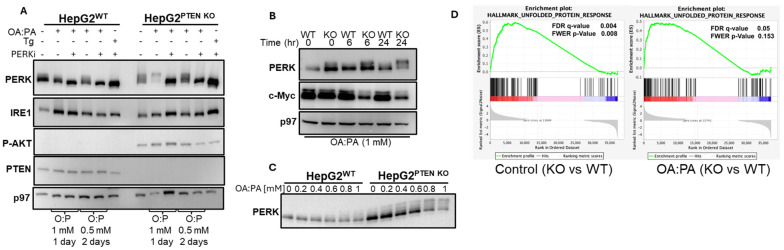
Deficiency of PTEN in hepatocytes generates hyperactivation of the UPR in response to a mixture of OA and PA. (**A**). HepG2 wt controls and PTEN KO were treated with a mixture of OA and PA at 1 mM for 1 day or 0.5 mM for 2 days. PERKi was added at 500 nM. The adhered live cells at the end of the treatments were isolated, and whole cell extracts were prepared and analyzed by immunoblotting to the different proteins. p97 was used as a loading control. (**B**). wt and PTEN KO HepG2 cells were treated with 1 mM OA:PA mixture for the indicated times and processed as in (**A**). (**C**). HepG2 wt controls and PTEN KO were treated for 24 h with a mixture of OA and PA at the indicated concentrations and processed as in (**A**). (**D**). Shown is the expression enrichment plot of UPR genes of PTEN KO relative to wt cells, as calculated from the RNA-Seq data.

HepG2 is a human hepatoma cell line that is commonly used to study HCC. Relative to other HCC cell lines, HepG2 cells express average levels of PTEN [37]. However, suppression of PTEN in HepG2 by siRNA activates the phosphorylation of AKT and mTOR and induces lipogenesis [38], rendering these cells suitable for our purposes. Using CRISPR mutagenesis, we generated PTEN-deficient HepG2 cells. The successful deletion was verified by immunoblotting for PTEN and for phosphorylated AKT, showing the expected loss of expression and gain of the phosphorylation phenotype. Control and PTEN KO cells were then treated with a 1:1 mixture of OA and PA, loaded on lipid-free BSA and added to the culture for up to 48 h. Unloaded BSA was used as the control. We used two concentrations of the lipid mixture, 0.5 and 1 mM. The 1 mM concentration was used for 1 day and the 0.5 mM for 2 days. Cells that were adhered to the culture dish were collected and assessed for the activation of the UPR by SDS-PAGE and immunoblotting. While ER stress conditions induce the expression of both IRE1 and PERK [39], the reduced electromobility of PERK via SDS-PAGE due to autophosphorylation is considered a more reliable indication of ER stress. In most experiments, we observed that PERK was moderately activated in PTEN KO cells under normal tissue culture conditions, along with a slightly higher expression. When cells were exposed to the lipids, PERK was activated preferentially in the PTEN KO cells, as indicated by the reduced electromobility pattern, which was sensitive to the inclusion of the PERK inhibitor (PERKi, GSK2606414) (Figure 1A). Based on the migration pattern of PERK, time and concentration dependence analyses indicated that ER stress in the PTEN KO cells requires approximately 24 h to develop, and at this time point, it reached a maximum with 1 mM of OA:PA (Figure 1B,C). To assess whether the PTEN KO HepG2 are inherently resistant to lipid-mediated ER stress, we treated the control and KO cells with cholesterol. An equal induction of PERK was observed in control and PTEN KO HepG2 cells (Figure S1A). This response was also observed when PA was added alone, without OA, a treatment reported to induce a stronger UPR than that when OA and PA are combined [40] (Figure S1B). When PTEN was re-expressed in the PTEN KO cells, PERK activation was reduced, as assessed by a smaller shift in electromobility (Figure S1C). To examine whether this phenomenon is specific to HepG2 cells, we knocked out PTEN in normal untransformed immortalized hepatocytes. A treatment with OA:PA generated ER stress predominantly in the PTEN KO cells (Figure S2), as was similarly observed for HepG2 cells. This indicates that PTEN deficiency predisposes the PERK pathway of hepatocytes, both normal and transformed, to free fatty acid-mediated UPR activation. To examine whether a canonical UPR occurs when treated by the OA:PA mixture, we conducted an RNA-Seq analysis for control conditions and following 1 mM OA:PA treatment for 24 h. Comparisons between treated and untreated wt cells showed an insignificant effect on the expression of genes in the adipogenesis and cholesterol homeostasis pathways, as well as in additional pathways. Unexpectedly, adipogenesis was reduced in the OA:PA-treated PTEN KO cells compared to the results for the control treatment, though not significantly. A marked suppression was noted for E2F target genes in the PTEN KO gene expression (Figure S3). When compared between cell types, a positive UPR signature was observed for PTEN KO relative to wt cells under normal culturing conditions. Although less significant, a positive UPR signature was maintained for PTEN KO versus wt HepG2 after OA:PA treatment (Figure 1D), indicating that PTEN KO cells mount a stronger UPR than wt cells in response to the OA:PA treatment. Of the three canonical UPR transducers, the PERK mRNA level was higher in the PTEN KO cells under control and treated conditions. IRE1 and ATF6 were less affected. However, downstream, bona fide targets of all three pathways were induced, indicating the activation of all UPR arms (Figure S4). We conclude that deficiency in PTEN predisposes HepG2 cells to activate the three canonical arms of the UPR in response to a mixture of monounsaturated and saturated fatty acids.

### 2.2. Deficiency of PTEN Generates a Terminal UPR in Response to a Mixture of OA and PA

**Figure 2:**
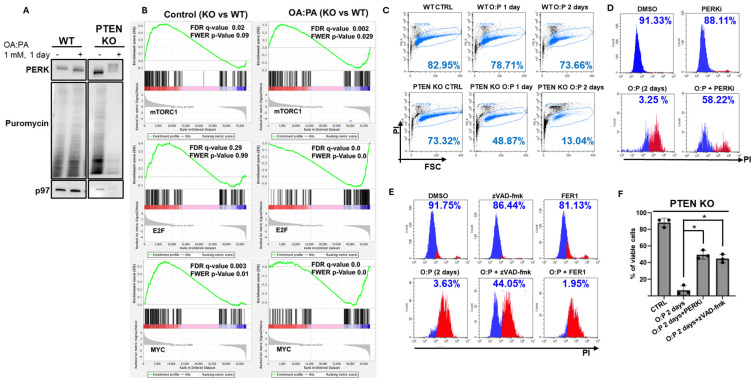
Deficiency of PTEN generates a terminal UPR in response to a mixture of OA and PA. (**A**). HepG2 wt and PTEN KO were incubated with OA:PA (1 mM, 24 h), washed, and puromycin was then added for 30 min. Cells were lysed, and whole cell extracts were prepared and analyzed by immunoblotting for puromycin. Shown is a representative outcome of three independent experiments. (**B**). Shown is the expression enrichment plot of genes of the mTORC1, E2F and MYC pathways in PTEN KO cells relative to that for wt cells, as calculated from the RNA-Seq data. (**C**). HepG2 wt and PTEN KO were incubated with 1 mM OA:PA for 24 or 48 h. Viability was determined by flow cytometry after incubation with propidium iodide. Shown are percentages of the live cells in one of three experiments. Quantification is shown in panel (**F**). (**D**). HepG2 wt and PTEN KO were incubated with 1 mM OA:PA for 48 h in the presence and absence of PERKi (500 nM). Shown is one of three experiments. Quantification is shown in panel (**F**). (**E**). HepG2 wt and PTEN KO were incubated with 1 mM OA:PA for 48 h in the presence and absence of zVAD-fmk (25 µM), or Ferrostatin 1 (25 µM). Shown is one of three experiments. Quantification of cell viability is shown in panel (**F**), which represents the percentage of propidium iodide-negative cells. Statistical significance was determined using an unpaired two-tailed Student’s *t*-test (* *p* < 0.05).

The UPR serves both cytoprotective and pro-death functions, depending on the magnitude and duration of the ER stress conditions [41]. Of the different pathways of the UPR, the PERK pathway is the most significant in directing cell death, primarily by apoptosis [42,43]. Recently, ferroptosis was connected to the hyperactivation of the IRE1 pathway [44]. PERK reduces protein synthesis by promoting the phosphorylation of eIF2α, which reduces translation initiation. To determine whether PERK is functionally active, we assessed protein synthesis by puromycin incorporation. Protein synthesis was strongly inhibited in the OA:PA-treated PTEN KO cells (Figure 2A). Analysis of the RNA-Seq data indicated that while under normal conditions, the pro-growth mTOR, E2F and Myc pathways were activated in PTEN KO relative to the results for the control cells, as expected from the absence of PTEN, these pathways were strongly inhibited in the PTEN KO cells following OA:PA treatment (Figure 2B). When assayed for viability, 48 h after the treatment, fewer PTEN KO cells survived relative to number of wt cells (Figure 2C, quantified in Figure 2F). The addition of PERKi significantly improved the survival of PTEN KO cells (Figure 2D, quantified in Figure 2F). Inclusion of the pan caspase inhibitor zVAD-fmk significantly reduced cell death, while the inhibitor of ferroptosis, ferrostatin 1, did not affect viability (Figure 2E, quantified in Figure 2F). We conclude that in the absence of PTEN, hepatocytes generate a terminal UPR in response to a mixture of OA and PA, which directs the cells to apoptosis.

### 2.3. The UPR Is Activated in PTEN KO HepG2 Cells in an AKT/mTOR-Independent Manner

**Figure 3:**
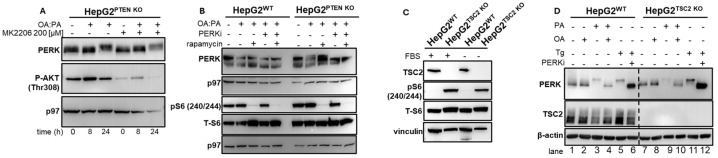
UPR is activated in PTEN KO HepG2 in an AKT/mTOR-independent manner. (**A**). PTEN KO HepG2 cells were treated with OA:PA (1 mM) for 8 or 24 h in the presence and absence of the allosteric AKT inhibitor MK2206. Cells were lysed, and whole cell extracts were prepared and analyzed by immunoblotting for PERK, P-AKT, and p97 as a loading control. Shown is a representative experiment of three. (**B**). HepG2 wt and PTEN KO were incubated with OA:PA (1 mM, 24 h). Tg (2.5 µg/mL), PERKi (500 nM) and rapamycin (100 nM) were added, where indicated. Cells were lysed, and whole cell extracts were prepared and analyzed by immunoblotting for PERK, Phospho-S6 (S240/244), total S6, and p97 as a loading control. Shown is a representative experiment of three. (**C**). wt and TSC2 KO HepG2 cells were incubated overnight with and without serum. Cells were lysed and whole cell extracts were prepared and analyzed by immunoblotting for the indicated proteins. Shown is a representative experiment of three. (**D**). wt and TSC2 KO HepG2 cells were treated with OA:PA (1 mM) for 24 h. Tg was added as a positive control. Cells were lysed, and whole cell extracts were prepared and analyzed by immunoblotting for PERK, TSC2, and β-actin as a loading control. Shown is a representative experiment of three.

PTEN is expressed in the cytoplasm and nucleus, where it displays multiple functions [45]. While the effect of PTEN inhibition on the PI3K/AKT/mTOR is prominent in cancer and has received most of the attention, PTEN affects additional signaling pathways, as well as directly regulates transcription by interacting with RNA pol II [46,47]. To determine whether ER stress develops in PTEN KO cells in an AKT/mTOR-dependent manner, we preincubated the KO cells with the allosteric AKT inhibitor MK2206 [48]. Notwithstanding the reduction in P-AKT levels, ER stress developed with similar kinetics (Figure 3A), nor did the addition of rapamycin, which allosterically blocks mTOR, affect the OA:PA-induced ER stress (Figure 3B). We then tested whether a direct activation of mTOR will recapitulate the phenotype of PTEN KO. Deletion of the inhibitory regulator of mTOR, TSC2, activates mTOR downstream of AKT in a manner insensitive to serum and other stress conditions [49] (Figure 3C). PA alone activated PERK, as observed in wt cells. When OA and PA were combined, modest activation of PERK was observed in TSC2 KO cells (Figure 3D, compare lane 4 to lane 10), although less than what was obtained when cells were treated with the ER stressor thapsigargin (Tg, 2.5 µg/mL, 8 h) and what has been observed for PTEN KO cells. We conclude that the hypersensitivity of the PTEN KO HepG2 cells to OA:PA is either independent of AKT/mTOR activity or that a transient inhibition of the AKT/mTOR does not reverse the hypersensitivity.

### 2.4. Peroxisome Content and β Oxidation Are Reduced in PTEN KO HepG2 Cells After OA:PA Treatment

**Figure 4:**
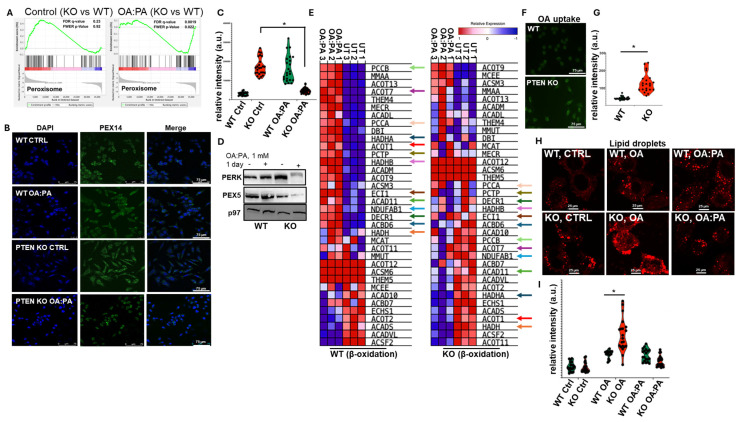
Peroxisome content is reduced in PTEN KO HepG2 after OA:PA treatment. (**A**). Shown is the expression enrichment plot of genes of the peroxisome pathway in PTEN KO relative to wt cells as calculated from the RNA-Seq data. Note the flip of the signature after the treatment with OA:PA. (**B**). Representative confocal immunofluorescence images for PEX14 before and after treatment with OA:PA. (**C**). Quantification of fluorescence intensity shows the reduction in PEX14 expression after treatment. Statistical significance was determined by an unpaired two-tailed Student’s *t*-test (* *p* < 0.05). (**D**). Immunoblotting for PEX5 before and after treatment with OA:PA in wt and PTEN KO HepG2 cells. Shown is a representative result of three repetitions. (**E**). Analysis of RNA-Seq data for the mitochondrial β oxidation pathway following OA:PA treatment. In arrows (each gene marked in a different color) are genes whose expression is upregulated in wt cells and downregulated in PTEN KO cells. (**F**). Uptake of OA in wt and PERK KO HepG2 cells. Fluorescence was calculated for 20 cells and quantified. (**G**). Statistical significance was determined using an unpaired two-tailed Student’s *t*-test (* *p* < 0.05). (**H**). Representative images of lipid droplets using LipidSpot in wt and PTEN KO HepG2 cells after incubation with OA and OA:PA for 24 h. (**I**). Quantification of 20 cells for each treatment. Statistical significance was determined using an unpaired two-tailed Student’s *t*-test (* *p* < 0.05).

We hypothesized that ER stress develops in PTEN-deficient hepatocytes due to an aberrant lipid metabolism, when cells are exposed to high concentrations of free fatty acids. β-oxidation occurs primarily in the mitochondria and peroxisomes. Both mitochondrial and peroxisome β oxidation activities are induced by free fatty acids due to the activation of Peroxisome Proliferator-Activated Receptor α (PPARα) to mitigate lipid-mediated toxicity [50,51]. Analysis by RNA-Seq indicated that under regular culturing conditions, peroxisome genes were upregulated in PTEN KO HepG2 cells; however, suppression develops following OA:PA treatment (Figure 4A). PEX14 is an essential protein to maintain peroxisome content and function. Assessment of peroxisome content by immunofluorescence to PEX14 confirmed the reduction in the PTEN KO cells following OA:PA treatment (Figure 4B, quantified in Figure 4C). The levels of a second peroxisome protein, PEX5, were also reduced (Figure 4D), in agreement with the immunofluorescence results. This was in contrast to a modest increase in peroxisome levels in both wt and PTEN KO HepG2 cells after treatment with OA (Figure S5). Analysis of the mRNA levels of mitochondrial β oxidation genes indicated a reversed response of wt and KO cells. While most genes were induced in the wt cells, downregulation of expression was noted for the PTEN KO cells (Figure 4E). The presumable reduction in fatty acid catabolism in the presence of high concentrations of exogenous OA and PA suggests that lipids accumulate more rapidly and to higher concentrations in the PTEN KO cells, assuming they penetrate at similar levels. To assess fatty acid uptake, we used a fluorescently tagged OA (TopFluor™ OA). Consistent with what was shown for primary mouse hepatocytes [52], the uptake of OA was higher in PTEN KO HepG2 cells than in the controls (Figure 4F, quantified in Figure 4G). We then estimated the ability of the cells to store the lipids in lipid droplets (LDs). LD content was assessed by staining the cells with LipidSpot 610 dye. When BSA loaded with OA alone was added to the cells, staining was higher in PTEN KO cells than in the controls. However, when OA was combined with PA, both cell types were stained at similar levels (Figure 4H, quantified in Figure 4I). These data suggest that in the presence of the OA:PA combination, despite higher intracellular concentrations of the lipids, PTEN KO cells do not produce large lipid droplets, as opposed to when they are exposed to OA alone. This may direct the lipids into other compartments, such as the ER, potentially generating ER stress.

### 2.5. PTEN KO HepG2 Cells Generate More TG and Less PC than Control Cells upon Treatment with OA:PA

**Figure 5:**
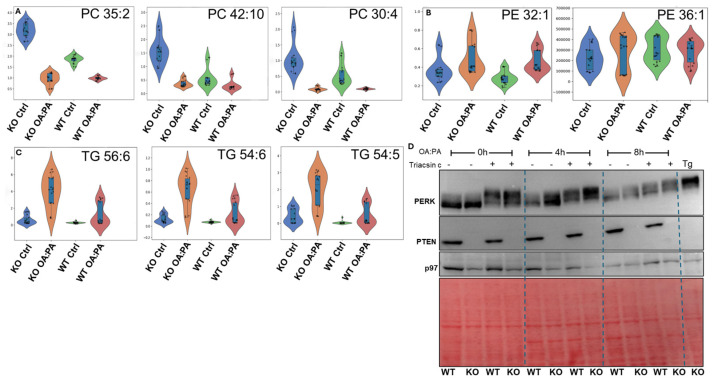
PTEN KO HepG2 cells generate more TG and less PC than do control cells upon treatment with OA:PA. Shown are lipidomic analyses of (**A**) phosphatidylcholine that contains OA or PA; (**B**) phosphatidylethanolamine that contains OA or PA; and (**C**) triglycerides that contain OA or PA. (**D**) Cells were treated with or without Triacsin C (2.5 µM) for 8 h alone or together with OA:PA for the indicated times. Tg treatment (2.5 µg/mL, 8 h) was used as a positive control. Shown is a representative immunoblot of three repetitions. p97 and β-actin were used as a loading control.

To capture global lipid homeostasis, we conducted a lipidomic analysis of control and PTEN KO HepG2 cells before and after treatment with OA:PA. Profound differences for many lipids species were found. Analyzing the lipidomic data indicated that PTEN KO cells under control conditions contained higher levels of phosphatidylcholine. However, the levels sharply decreased upon OA:PA treatment, particularly in phosphatidylcholine species that contain OA or PA. Phosphatidylcholine species that did not contain the exogenously provided lipids were less affected (Figure 5A). Because the relative ratio between phosphatidylcholine and phosphatidylethanolamine was shown to affect the SERCA pump and confer ER stress if reduced [53], we analyzed the levels of phosphatidylethanolamine species that contain OA or PA. For most, there was no difference (Figure 5B), suggesting that the ratio of phosphatidylcholine to phosphatidylethanolamine drops in response to OA:PA treatment in the PTEN KO cells. An increase in triglycerides that contain OA or PA was observed in the PTEN KO cells (Figure 5C). We then analyzed the RNA-Seq data for the TG and PC metabolic pathways. We found specific genes that were oppositely regulated in wt and PTEN KO HepG2 cells. Consistent with the reduced LD biogenesis in PTEN KO cells we found a reduced expression of DGAT2 (Figure S6A,B), an enzyme required for the synthesis of TG [54]. Analysis of PC biogenesis also revealed a few genes that were oppositely regulated in both wt and PTEN KO HepG2 cells. One of these is phosphatidylethanolamine N-methyltransferase (PEMT), which converts PE to PC. Deficiency of PEMT alters hepatic phospholipid composition and induces ER stress [55]. PEMT was upregulated in wt and downregulated in the PTEN KO cells following OA:PA treatment, suggesting a role in the hypersensitivity of PTEN KO cells to lipid toxicity (Figure S6C,D). Based on these findings, we suggest that exposure of PTEN KO HepG2 cells to a mixture of OA and PA results in the routing of the lipids into triglycerides more than into phospholipids and the inhibition of PC biogenesis. The triglycerides are probably stored in small droplets that were not detected by fluorescent staining for LD. We suggest that these rapid changes in lipid homeostasis trigger the UPR. To determine whether the perturbation of TG homeostasis promotes ER stress, we treated the cells with the TG biosynthesis inhibitor, Triacsin C. Based on the electromobility of PERK, a pretreatment for 12 h with Triacsin C induced ER stress to a similar level in both PTEN KO and control cells. The stress was stronger than that derived from a treatment with OA:PA for up to 8 h (Figure 5D). We conclude that in HepG2 cells, the formation of TG protects the cells from OA:PA-induced ER stress and further supports the metabolism of free fatty acids as the reason for enhanced ER stress in PTEN KO cells.

### 2.6. A Diet Rich in Saturated Fatty Acids Retards the Progression of PTEN KO Hepatoma Cells

**Figure 6:**
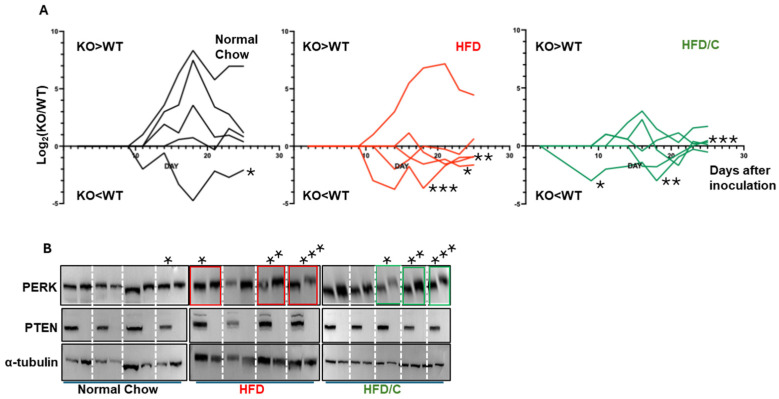
A diet rich in saturated fatty acids retards the progression of PTEN KO cells relative to that of wt HCC. Immunocompromised NSG mice were placed on the indicated diets for 4 weeks and then challenged with PTEN KO and wt HepG2 on opposite flanks. Tumor growth kinetics are presented in Figure S5. (**A**). The ratio of tumor volumes of KO and wt cells was logarithmically transformed. Positive values represent a tumor volume of PTEN KO larger than that of wt, while the opposite is true for negative values. (**B**). At the end of the experiment, tumors were extracted, lysed, and analyzed by immunoblotting to PERK, PTEN and α-tubulin as a loading control. Individual mice that display activation of PERK are marked by asterisks. Shown in (**A**) are their relative growth curves.

The development of ER stress preferentially in PTEN KO cells suggests a potential use for therapy. To determine whether this approach can curtail the growth of PTEN KO HCC in vivo, immunocompromised NSG mice were placed on a normal diet, a conventional high-fat diet (HFD), or a HFD supplemented with extra sugar and cholesterol (HFD/C). After four weeks of ad libitum feeding, before the mice became obese, wt HepG2 cells were implanted under the skin of the right flank, and PTEN KO HepG2 cells were implanted under the skin of the left. Tumor growth was measured with a caliper three times a week. As observed for the individual mice, on a normal diet, PTEN KO tumors progressed faster than did the control cells in three out of five mice. This trend was reversed when mice were fed with a HFD. Under the HFD/C diet, both tumors progressed at similar rates (Figure S7). In one mouse of the control diet and one mouse of the HFD cohorts, the wt tumor did not develop. Of note, for the HFD group, this was the only incident where the PTEN KO tumor grew faster than the wt tumor, which may be a consequence of a technical issue rather than a true biological outlier. To better visualize the relative growth kinetics, we plotted the ratio between the PTEN KO to wt tumor size over time for the individual mice using a logarithmic scale. Positive values reflect a larger PTEN KO tumor, whereas negative values reflect a larger wt tumor in the same host. More values were positive in the normal diet cohort (black lines). More values were negative when mice were fed with the HFD (red lines). Under the HFD/C diet, the relative growth patterns were evenly distributed in both sides of the abscissas (green lines) (Figure 6A). We then extracted and lysed the tumors from each mouse and analyzed ER stress by assessing the migration of PERK on SDS-PAGE. Missing are the pairs in which the wt cells did not grow. Therefore, immunoblotting is shown for only four mice in the control and HFD groups. We observed evidence of ER stress in the PTEN KO tumors in several of the mice (boxed, Figure 6B). Intriguingly, in all these individual mice, the growth of the KO tumor was retarded relative to that of the wt control, including in one of the mice fed on the control diet (labeled by stars in Figure 6A). We conclude that by providing a diet rich in lipids, it is possible to selectively inhibit the tumorigenicity of PTEN KO HCC. However, the optimal diet should be rationally designed.

## 3. Discussion

Conditional deletion of PTEN in the liver using the albumin promoter generates steatosis in almost all hepatocytes at 6 months of age, accompanied with decreased body fat content [52]. This condition progresses to HCC within 9–12 months in almost 100% penetration. Steatosis has been attributed to enhanced lipid biosynthesis, driven by the activation of the AKT/mTOR pathway. Intriguingly, the HCC cells in this mouse model, while still PTEN-deficient, are largely not steatotic [56]. The absence of steatosis in HCC suggests that in order to support proliferation, cancer cells employ strategies to mitigate the accumulation of LDs, despite an a priori pro-steatotic program. We found that in doing so, PTEN-deficiency in HCC cells become predisposed to develop a strong terminal UPR when fatty acids are exogenously provided. This vulnerability is selective to certain free fatty acid combinations, as control and PTEN KO cells were equally sensitive to cholesterol- and PA-only-mediated ER stress. It should be noted that the induction of ER stress requires mM concentrations and a long incubation time, suggesting the involvement of metabolism (Figure 1 and Figure 2). This is leveraged by the intrinsically high metabolic activity of hepatocytes, as evident by the strong ER stress that develops in the presence of a TG biosynthesis inhibitor, such as triacsin C, under normal tissue culture conditions.

Death of PTEN KO HepG2 cells by OA:PA is by apoptosis, driven in part by the PERK pathway of the UPR. We propose that the PTEN deficiency generates an imbalance between the uptake of fatty acids and their storage in large LDs (Figure 4). This imbalance promotes the incorporation of the exogenous lipids into membranes, including that of the ER. Ferrostatin-1 did not improve viability, indicating a minimal contribution of ferroptosis, which may be explained by the inhibitory effect of OA on lipid peroxidation [57]. In addition to enhanced cell death, PTEN KO cells respond to OA:PA by suppressing the E2F and Myc oncogenic pathways, suggesting a cytostatic effect. We previously described a connection between ER stress and E2F1 suppression through the involvement of microRNAs; however, in this context, the IRE1 pathway was central [58]. We therefore invoke the involvement of a different mechanism, which is currently unknown. We suggest that this strategy, which generates a combined apoptosis and suppression of oncogenic pathways, can be leveraged into therapy of an HCC subtype that displays clinically limited therapeutic options.

Which upstream mechanisms contribute to the selectivity of ER stress in the PTEN-deficient hepatocytes? In most cell types, the deletion of PTEN mildly activates AKT, owing to the negative feedback from mTOR [59]. Gain of function and loss of function analyses of the PI3K/AKT/mTOR pathway ruled out mTOR activation as the main underlying cause (Figure 3). In addition, the treatment with OA:PA caused a reduction in P-AKT levels (Figure 1). Therefore, we suggest the involvement of AKT-independent mechanisms, which are poorly defined and are dependent on the cellular context [60]. For instance, in fibroblasts, PTEN deficiency activates cyclooxygenase2 through a poorly understood pathway [61]. In epithelial cells, the loss of PTEN activates β catenin in a GSK3β-dependent manner [62]. In liver cells, PTEN is a major metabolic modulator that affects glycolysis, gluconeogenesis, glycogen synthesis and lipid metabolism, as well as mitochondrial metabolism [63]. In its absence, livers may undergo an irreversible metabolic rewiring that cannot be reversed by AKT inhibition (Figure 3). Of note, even the re-expression of PTEN only partially alleviated the activation of stress by OA:PA (Figure S1), suggesting that the deletion of PTEN may cause cellular plasticity. We propose that this plasticity endows HCC with protection against the steatosis driven by intrinsic lipid biosynthesis. However, when flooded by exogenous lipids, toxicity develops more readily. We underscore a kinetic defect in LD growth, which also affects the PC/PE ratio (Figure 5), most likely resulting in the activation of the UPR. To further elucidate the molecular details of this process, ER membranes should be isolated and analyzed for lipid content in a time-dependent manner.

The reduction in peroxisome content and expression of genes in the β oxidation pathway in PTEN KO HepG2 cells was unexpected. Normally, cells respond to fatty acids by activating peroxisome biogenesis. According to the RNA-Seq analysis, the mRNA levels of PPARα, a central regulator of peroxisome biogenesis [64,65], were not significantly affected by the treatment. mRNA levels of other regulators of peroxisome biogenesis markers such as PPARγ and PGC-1α were mildly reduced. Though demonstrated in yeast, peroxisome biogenesis requires a condensate of PEX14 and PEX13 to initiate the process [66]. An intriguing possibility for the reduction in peroxisome content can be the consequence of perturbed protein phase separation, triggered by the aberrant lipid trafficking in PTEN KO cells. Alternatively, the inhibition of peroxisome biogenesis can occur downstream of ER stress, rather than cause it. We conjecture that the reduction in peroxisome content amplifies the effect of OA:PA in the PTEN KO cells by reducing lipid catabolism and enforcing the elongation of the exogenously provided fatty acids. The use of isotopes labeled OA and PA can help to clarify the mechanism.

More than half of HCC patients are diagnosed at an advanced stage. The common drug therapy is a combination of immune checkpoint inhibitors with tyrosine kinase inhibitors. Other intervention modalities are determined in accordance with disease stage, the etiology of HCC, and patient status [67]. Hence, the common practice of HCC therapy does not typically involve personalized treatment, primarily because of the lack of a clear driver mutation that is considered clinically impactful for therapy. As a result, liver biopsies are not typically acquired [68]. When PTEN KO mice are fed with a HFD, steatosis is exacerbated, while endogenous lipid biosynthesis is reduced [69]. Enforced ER stress in the liver by a co-deletion of BiP and PTEN accelerates steatosis and promotes the transformation to HCC. Intriguingly, when the HCC cells were analyzed, BiP was expressed, and ER stress was quelled [36]. These findings suggest that ER stress in the background of PTEN deficiency is a driver for transformation into cancer. However, the cells that eventually transform are those able to overcome the stress, reinforcing the notion that ER stress, together with steatosis, is a liability for cancer progression. Our data suggest that these basic features can be utilized for therapy, provided that a high concentration of fatty acids in the right balance between saturated and non-saturated fatty acids are supplied. Consistent with this model is the in vivo progression of PTEN KO HepG2 cells in the presence of HFD (Figure 6). It should be emphasized that immediately after absorption, medium- and long-chain free fatty acids are packed as triglycerides in chylomicrons. Therefore, when free fatty acids at the size of OA or PA are to be added to the diet, only a small fraction will accumulate in the circulation as free acids, while the majority of these lipids will accumulate as triglycerides in adipose tissues, requiring lipolysis to reach the circulation. Short-chain fatty acids up to six carbons long are readily absorbed as is. It remains to be tested whether natural short-chain fatty acids such as caproic acid C (6:0) or crotonic acid C (4:1) can elicit the preferential ER stress in PTEN KO HCC, as obtained with the OA:PA combination. If this sensitivity necessitates long-chain fatty acids for therapeutic purposes, we anticipate that treatments with albumin loaded with lipids will be a better approach than their use as dietary supplements. We suggest that if these findings represent a general metabolic susceptibility, the status of PTEN expression in the tumors should be determined and taken into account for simple dietary recommendations that are counterintuitive to the current common practice. Namely, increasing the consumption of certain fats may be beneficial to HCC patients in which PTEN expression was compromised. The specific fatty acids, their exact amounts, and their best mode of administration, orally or parenterally, require additional testing.

## 4. Materials and Methods

*Cell culture*: HepG2 and 293T cells were originally purchased from ATCC (Manassas, VA, USA). *PTEN* KO HepG2 cells were previously described in [70]. Immortalized primary mouse hepatocytes were provided by Dr. Angela Valverde (Instituto de Investigaciones Biomédicas Sols-Morreale, Madrid, Spain). Cells were maintained in DMEM media supplemented with 10% FBS, Sodium Pyruvate 1%, Sodium Glutamate 1% and Pen/Strep 1%. All media additives were purchased from Thermo Fisher Scientific (Waltham, MA, USA).

*DNA transfection*: Cells were first plated in a fresh medium at a confluency of 60–70% for 24 h. Then, a plasmid of interest was resuspended in Opti-MEM medium (Thermo Fisher Scientific, 31985062; Waltham, MA, USA) and incubated for 10 min, then thoroughly mixed with X-tremeGENE™ 360 Transfection Reagent (Millipore Sigma, X-tremeGENE; St. Louis, MO, USA) at ratio of 1 μg to 3 μL, then incubated for 20 min. This mixture was positioned dropwise on the plated cells, which were cultured thereafter for an additional 24–72 h, with fresh medium replacement every 24 h.

*Lentiviral transduction*: HEK293T cells were transfected with the lentiviral transfer plasmid of interest, along with the lentiviral packaging plasmid pCMV-dR8.2 dvpr (addgene #8455) and the envelope plasmid pCMV-VSV-G (addgene #8454), at ratio of 3:2:1. Lentiviral-containing medium was collected 48 and 72 h post transfection, filtered through 0.45 μm (Cytiva, 10462100; Marlborough, MA, USA), and either used directly or frozen at −80 °C for future use. Cells to be transduced were plated to reach a 50–60% confluency, then lentiviral-containing medium was added, along with Polybrene (Millipore Sigma, TR-1003-G; St. Louis, MO, USA) at final concentrations of 33.34% and 10 μg/mL, respectively. Transduced cells were selected after 24 h of a 72 h treatment with 0.5–1 μg/mL puromycin.

*Gene editing using CRISPR-Cas9 system*: Cas9, along with its single-guided RNA (sgRNA), were introduced to cells via lentiviral infection using lentiCRISPRv2 hygro (Addgene #98291; Watertown, MA, USA) [71]), followed by selection with Hygromycin (50 µg/mL). sgRNAs targeting genes of interest were designed using the Broad Institute portal CRISPick (https://portals.broadinstitute.org/gppx/crispick/public, accessed on 11 March 2026) [72,73]. Human *TSC2* gRNA: 5′ TCT TCG TAG GGA TGG CAC TC 3′; Mouse *PTEN* gRNA: 5′ ACT TTG ATA TCA CCA CAC AC 3′. To generate complete gene knockout (KO), cells were further seeded at one cell per well in a 96-well plate. Single-cell colonies were expanded and screened by immunoblotting of the targeted protein.

*OA:PA treatment*: Sodium oleate stock was dissolved in DDW to 10 mM in DDW at 50 °C. After obtaining a clear solution, the sample was diluted 1:10 in serum-free DMEM with 1% lipid-free BSA at 37 °C. Sodium palmitate stock solution was prepared similarly to 10 mM in DDW containing 11% lipid-free BSA and diluted 1:10 in serum-free DMEM with 1% delipidated BSA. Normal growth media was aspirated and replaced with the OA:PA mixture. Triacsin C (HY-N6707, MedChemExpress; Monmouth Junction, NJ, USA) was dissolved in DMSO to 5 mM and added to the cells at 1:2000 dilution.

*Uptake of fluorescent OA*: HepG2 cells were seeded on glass coverslips at a confluency of approximately 60–70% and cultured for 24 h. Cells were then incubated for 3 h with TopFluor Oleic Acid (Avanti Polar Lipids—810259C-1MG-F-010; San Diego, CA, USA) at a final concentration of 0.1 mg/mL. Following incubation, cells were washed three times with PBS and subsequently fixed with 4% paraformaldehyde for 20 min at room temperature. After fixation, cells were washed three additional times with PBS and mounted using mounting medium containing DAPI (Vector Laboratories–WOVUS35055; Newark, CA, USA).

*Measurement of cell viability*: Cells were seeded in fresh culture medium at a confluency of approximately 70–80% and incubated for 24 h. Subsequently, cells were treated with the indicated lipid mixtures for 24 or 48 h. Following treatment, both adherent and floating cells were collected and washed twice with PBS. The cell pellets were then resuspended in PBS containing 5% fetal bovine serum (FBS). Propidium iodide (PI; Millipore Sigma—No. P4170; St. Louis, MO, USA) was added immediately before analysis to stain nonviable cells. Fluorescence intensity was then measured using a flow cytometer.

*Immunoblotting*: Cells were collected, washed with PBS and centrifuged at 4000× *g* and 4 °C for 3 min. Cell pellets were either frozen at −80 °C for future lysis or lysed directly using ice-cold lysis buffer (50 mM Tris-HCl pH = 7.5, 150 mM NaCl, 0.2% CHAPS, 0.1% SDS, 1% IGEPAL CA-630) supplemented with proteases and phosphatases inhibitors (TargetMol, C0001, C0002 and C0003; Wellesley Hills, MA, USA). Lysates were incubated on ice for 10 min, then centrifuged at 19,000× *g* and 4 °C for 15 min. Produced supernatants were then separated, quantified, and mixed with sample buffer (180 mM Tris-HCl pH = 6.8, 7% SDS, 30% glycerol, 400 mM DTT, 0.003% bromophenol blue) at ratio of 3:1. Samples were denatured by heat (95 °C for 5 min) and then loaded onto SDS-polyacrylamide gel (SDS-PAGE) fixed within 1.5 mm spaced glasses, submerged in running buffer (25 mM Tris, 192 mM glycine, 0.1% SDS), and then resolved by electrophoresis (120–150 V for 1–1.5-h). Proteins in the SDS-PAGE were then transferred onto a PVDF/nitrocellulose membrane by electrophoresis (100 V for 1.5 h) in transfer buffer (25 mM Tris, 192 mM glycine, 10% methanol). The blotted membrane was blocked with 5% skim milk (BD Life Sciences, 232100; Franklin Lakes, NJ, USA) dissolved in TBST buffer (10 mM Tris-HCl pH = 8, 150 mM NaCl, 0.1% Tween) for 1 h at RT and then washed three times with TBST. The blocked membrane was incubated with primary antibody solution (0.01% antibody in 5 mL TBST and 0.1% NaN_3_) in a rolling tube overnight at 4 °C. The membrane was then washed three times with TBST and incubated with Horseradish peroxidase (HRP) conjugated secondary antibody for 1 hour at RT, washed again, and then detected by chemiluminescence using Bio-Rad ChemiDoc™ XR (Hercules, CA, USA). Immobilon^®^ Crescendo (Millipore Sigma, WBLUR0500; St. Louis, MO, USA) was used as the chemiluminescent substrate. The antibodies that were used are listed here: anti-PERK (CST #3192), anti-IRE1 (CST #3294), anti-pAKT (CST #13038), anti-PTEN (CST #9188), anti-Puromycin (Millipore Sigma, MABE341; St. Louis, MO, USA), anti-cMYC (abcam #ab32072), anti-P-S6-S240/244 (CST #5364), anti-S6 (CST #2217), anti-TSC2 (CST #4308), and anti-PEX5 (CST #83020). For loading controls, we used anti-β-actin (CST #4970), anti-p97 (kindly provided by Dr. Ariel Stanhill, the Open University, Israel), and anti-vinculin (CST #13901). Secondary HRP-conjugated antibodies comprised goat anti-mouse (Jackson ImmunoResearch, 115-035-003; West Grove, PA, USA) and goat anti-rabbit (Jackson ImmunoResearch, 111-035-003; West Grove, PA, USA) antibodies.

*Immunofluorescence and lipid droplet staining*: Cells were seeded on glass coverslips and cultured for 24 h. Subsequently, cells were treated with indicated lipid mixtures for 24 h. Following treatment, cells were fixed with 4% paraformaldehyde (PFA) for 20 min at room temperature and washed three times with PBS. Permeabilization was performed using 0.2% Triton X-100 in PBS for 5 min at room temperature, followed by three PBS washes (3 min each). Cells were then incubated with blocking buffer (3% BSA in PBS) for 1 h at room temperature. After blocking, cells were incubated with the primary antibody PEX14–Alexa Fluor 488-conjugated (Proteintech—CL488-10594; Rosemont, IL, USA) for 2 h at room temperature. Samples were subsequently washed three times with PBS (5 min each). For lipid droplet visualization, fixed cells were incubated with 1× LipidSpot™ 610 Lipid Droplet Stain (Biotium-—70069-T; Fremont, CA, USA) for 20 min at room temperature, followed by three PBS washes. Finally, coverslips were mounted using mounting medium containing DAPI (Vector Laboratories, Cat. No. WOVUS35055; Newark, CA, USA), and images were acquired using a Leica SP8 confocal microscope (Wetzlar, Germany).

*RNA sequencing and analysis*: Control HepG2 and PTEN KO HepG2 were treated in DMEM media supplemented with empty BSA or 1 mM OA:PA BSA. Total RNA samples for three biological replicates (approximately 1 × 10^6^ live cells) were subjected to sequencing. Raw reads were processed for quality trimming and adaptors removal using fastx_toolkit v0.0.14 and cutadapt v2.10. The processed reads were aligned to the human transcriptome and genome version GRCh38 with annotations from Ensembl release 106 using TopHat v2.1.1. Counts per gene quantification were obtained with htseq-count v2.01. Normalization and differential expression analysis were done with the DESeq2 package v 1.36.0. Pair-wise comparisons were tested with default parameters (Wald test), without applying the independent filtering algorithm. The significance threshold was taken as padj < 0.1. In addition, significant DE genes were further filtered by the log2FoldChange value. This filtering was baseMean-dependent and required a baseMean above 5 and an absolute log2FoldChange higher than 5/sqrt (baseMean) + 0.3 (for highly expressed genes, this means the requirement for a fold change of at least 1.2, while genes with a very low expression would need a 5.8-fold change to pass the filtering).

*Lipidomic data analysis*: HepG2 cells were seeded and cultured for 24 h, followed by treatment with the indicated lipid mixture for 24 h. After treatment, adherent cells were collected, and the cell pellets were washed once with PBS. The washed pellets were immediately flash-frozen in liquid nitrogen and stored at −80 °C. Lipids were extracted by immersing the cell pellet in 250 μL of analytical grade methanol, followed by six consecutive freeze–thaw cycles in liquid N2, with a round of 15 min of sonication in an ice-water bath on the 3rd and 6th cycle. The samples were then centrifuged for 15 min at 15 K rpm (16,602 cf) at 4 °C. The supernatants were collected and further passed through a 0.22 micron spin filtering column (Costar spin-x filters) at 10 K rpm for 10 min at 4 °C. The final filtrated was transferred to 1.5 mL autosampler vials with a 150 μL insert, capped, and subjected to LC/MS lipidomics profiling. The MS data were acquired with a Thermo Scientific IDX Hi-Res mass analyzer system using the AcquieX acquisition workflow (data dependent analysis). The MS operating parameters were as follows: MS1 mass resolution 120 K; MS2 30 K scan range 250–1250; stepped energy (HCD) 23,25,27; RF len (%) 45; AGC gain; intensity threshold 2 × 10^4^ 25% custom injection mode with an injection time of 54 ms. The chromatographic separations were archived on a Thermo Scientific Vanquish Flex system using a Waters Acquity UPLC CSH C18 column (2.1 × 150 mm with a particle size of 1.7 μm) (Milford, MA, USA), operating at 55 °C with flow rate of 250 μL/min. The LC gradient consists of a binary buffer system, i.e., Buffer A (LC/MS grade water/ACN 40/60 *v*/*v*) and Buffer B (90:10 IPA and ACN), with both buffers containing with 10 mM ammonium formate. Independent buffer systems were used for positive and negative mode acquisition, respectively; for positive mode, the pH of buffers was adjusted using 0.1% formic acid and for negative mode, 0.1% ammonia solution was used. The LC gradient was the same for both polarities: 60% B at T0, hold for 1.5 min, and linearly increase to 85%B at 7 min; then increased to 95%B at 12.5 min, hold for 4.5 min, return to starting condition, and hold for further 4.5 min (column stabilization). The injection volume used for the positive mode was 2 μL and for negative mode, it was 3 μL, respectively. The voltage applied for the positive mode and negative modes was 3.5 kV and 2.5 kV, respectively. The HESI conditions for 200 μL were as follows: sheath gas, 35; aux gas, 7; and sweep gas, 0. The ion transfer tube temp was 300 °C, and the vaporizer temp was 275 °C. The lipidomic pos and neg datasets were processed independently via the Thermo scientific Lipid search version 4 workflow: HCD (high energy collision database) retention time (0.1 min), parent ion mass tolerance (5 ppm), and product ion (10 ppm). The alignment method (max) was as follows: top rank off, minimum m-score 5.0, all isomer peaks, and ID quality filter A and B only. Lipid IDs were matched using the lipid search in the silico library MS2 level, with an MS/MS similarly match of 80% or higher. Quality control: Corresponding lipidomics pooled QCs samples were used to assess for instrumental drifts; the RSD variation across the QCs for HILIC and lipidomics assessment were less than 15%, respectively. Any metabolite/lipid features which had an RSD of 25% or less within the QCs were retained; this is extended to the rest of the dataset. All results and visualizations were generated using Metaboanalyst 6.0. The dataset was quantile normalized, log 2 transformed, and auto scaled prior to multivariate analysis and interpretation.

*In vivo analysis of tumor growth*: Studies were performed in accordance with recommendations in the Guide for the Care and Use of Laboratory Animals of the National Institutes of Health and were conducted in the Athymic Animal & Xenograft Core Facility under an IACUC protocol approved by the Case Comprehensive Cancer Center Preclinical & Translational Shared Resource. Eight-week-old NSG-SGM3-IL15 male mice were purchased from Jackson Laboratories. Cohorts of five mice each were placed on a regular chow diet, a high-fat diet (HFD, diet# TD.07011, Inotiv; West Lafayette, IN, USA), or a high-fat diet enriched with cholesterol and sugar (HFD/C, diet# TD.190142, Inotiv). After four weeks, 1 × 10^6^ of live HepG2 or PTEN KO HepG2 cells suspended in 150 µL of PBS were inoculated subcutaneously to the right and left flank of each mouse, respectively. Measurements of tumor volumes were obtained three times a week, and the mice were sacrificed when the tumor size exceeded 2 cm of a single dimension or 4000 mm^3^ in size, calculated by the V = 0.5 LW^2^, in which L is the largest of the two dimensions.

## 5. Conclusions

Our data suggest that when PTEN-deficient hepatocytes transform into HCC, a metabolic remodeling occurs that limits intracellular steatosis, which is needed to support cell proliferation. Mechanistically, we suggest that the reduction in lipid catabolism due to reduced peroxisome levels and the inhibition of LD formation are major components of the remodeling. This exposes a vulnerability to exogenously provided free fatty acids, which generates a terminal UPR. This vulnerability can be exploited for treatment and calls for the analysis of PTEN expression in HCC patients.

## Data Availability

The original contributions presented in this study are included in the article and Supplementary Materials. Further inquiries can be directed to the corresponding authors.

## Supplementary Materials

The following supporting information can be downloaded at: https://www.mdpi.com/article/10.3390/ijms27062778/s1.

## Abbreviations

The following abbreviations are used in this manuscript:

wt	Wild type
HCC	Hepatocellular carcinoma
UPR	Unfolded protein response
PTEN	Phosphatase and tensin homolog
ER	Endoplasmic reticulum
PIP2	Phosphatidylinositol (4,5)-bisphosphate
PIP3	Phosphatidylinositol (3,4,5)-trisphosphate
mTOR	Mechanistic target of rapamycin
OA	Oleic acid
PA	Palmitic acid
MAFLD	Metabolic dysfunction-associated fatty liver disease
MASH	Metabolic dysfunction-associated steatohepatitis
eIF2α	Eukaryotic translation initiation factor 2A
TSC2	Tuberous sclerosis complex 2
PERK	Protein kinase R-like endoplasmic reticulum kinase
PC	Phosphatidylcholine
PE	Phosphatidylethanolamine
IRE1	Inositol-requiring enzyme 1
TG	Triglycerides
ATF4	Activating transcription factor 4
ATF6	Activating transcription factor 6
XBP1	X-box binding protein 1
PEX14	Peroxin 14
PEX5	Peroxin 5
LD	Lipid droplet
