# Supplementary material for: Deficiency of PTEN Confers Hypersensitivity to Fatty Acid-Mediated ER Stress in Transformed Hepatocytes
Source: Int J Mol Sci. 2026 Mar 19;27(6):2778. doi: 10.3390/ijms27062778 (PMC13027169; doi:10.3390/ijms27062778)
Supplement: Supplementary file 1 [file ijms-27-02778-s001.zip › Supplemental figures IJMS.pdf]

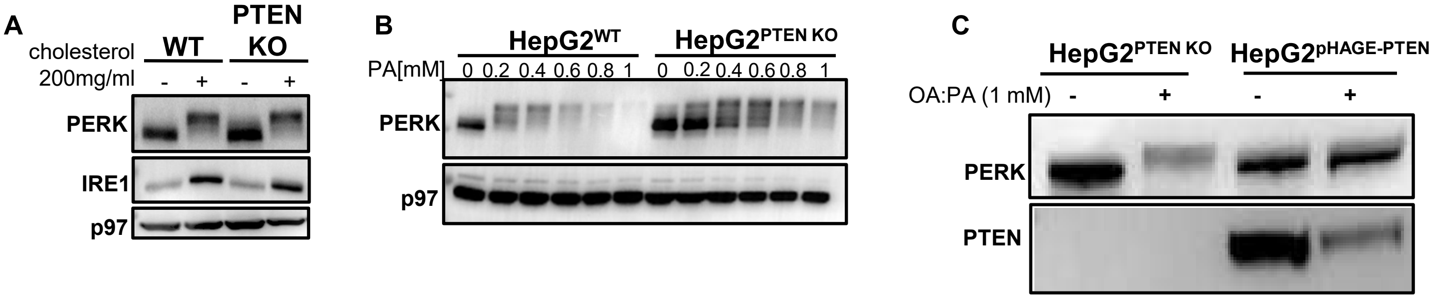

Fig. S1: Development of ER stress in hepatocytes. A. WT and PTEN KO were treated for 24 h with 200 mg/ml cholesterol loaded on cyclodextrin. Cells were lysed and whole cell lysates were analyzed by SDS-PAGE and immunoblot for the indicated proteins. Shown is a representative image of three independent repetitions. **B.** WT and PTEN KO were treated with the indicated concentrations of PA for 24 h. Cells were lysed and whole cell lysates were analyzed by SDS-PAGE and immunoblotting for the indicated proteins. Shown is a representative image of three independent repetitions. **C.** PTEN KO HepG2 cells were infected with lentiviruses that encode PTEN (Addgene, plasmid #116780), following by selection with hygromycin. Cells were then treated with OA:PA for 24 h and cells were lysed and whole cell lysates were analyzed by SDS-PAGE and immunoblotted for PERK and PTEN. Shown is a representative image of three independent repetitions.

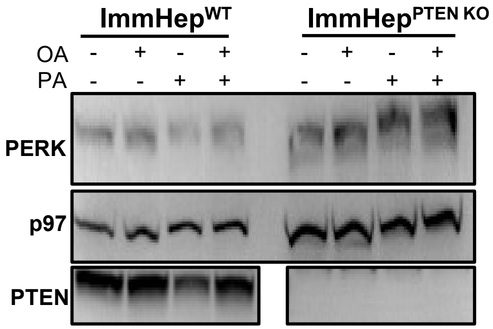

Fig. S2: ER stress in non-transformed hepatocytes. Large T immortalized primary murine hepatocytes were subjected to CRISPR/Cas9 mutagenesis to delete PTEN. KO was confirmed by two independent Western blotting. Cells were treated with OA, PA and combination of both (1 mM) for 24 h, lysed and whole cell lysates were analyzed by SDS-PAGE and immunoblotting for PERK and PTEN. p97 was used as a loading control. Shown is a representative image of three independent repetitions.

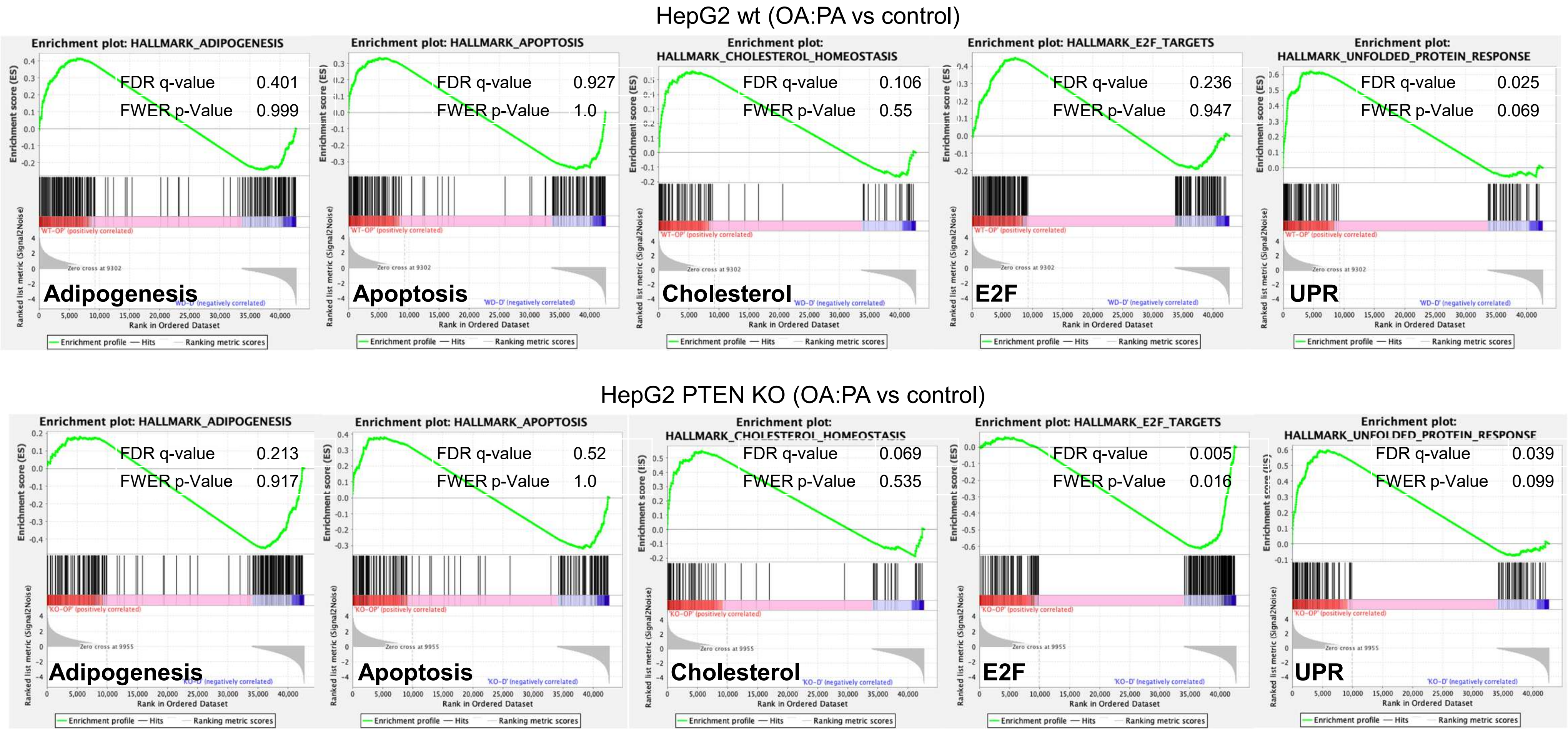

Fig. S3: Analysis of differentially expressed genes in wt and PTEN KO HepG2. Cells were treated with OA:PA for 24 h with a mixture of 1 mM of OA and PA in triplicates. RNA was extracted and subjected to RNAseq. Shown from left to right are the results for categories of Adipogenesis, Apoptosis, Cholesterol homeostasis, E2F targets and the UPR.

Fig. S4: All arms of the UPR are activated by OA:PA. Shown are the specific genes in the UPR signature and their relative expression in control and PTEN KO HepG2 under control and OA:PA treatment for 24 h. Labeled in black arrows are direct targets of XBP1, in blue arrows are direct targets of ATF6 and in red arrows are direct targets of ATF4. In purple are the proximal ER transducers PERK (EIF2AK3), IRE1 (ERN1) and ATF6. All genes were significantly induced in PTEN KO vs WT HepG2 following treatment with OA:PA .

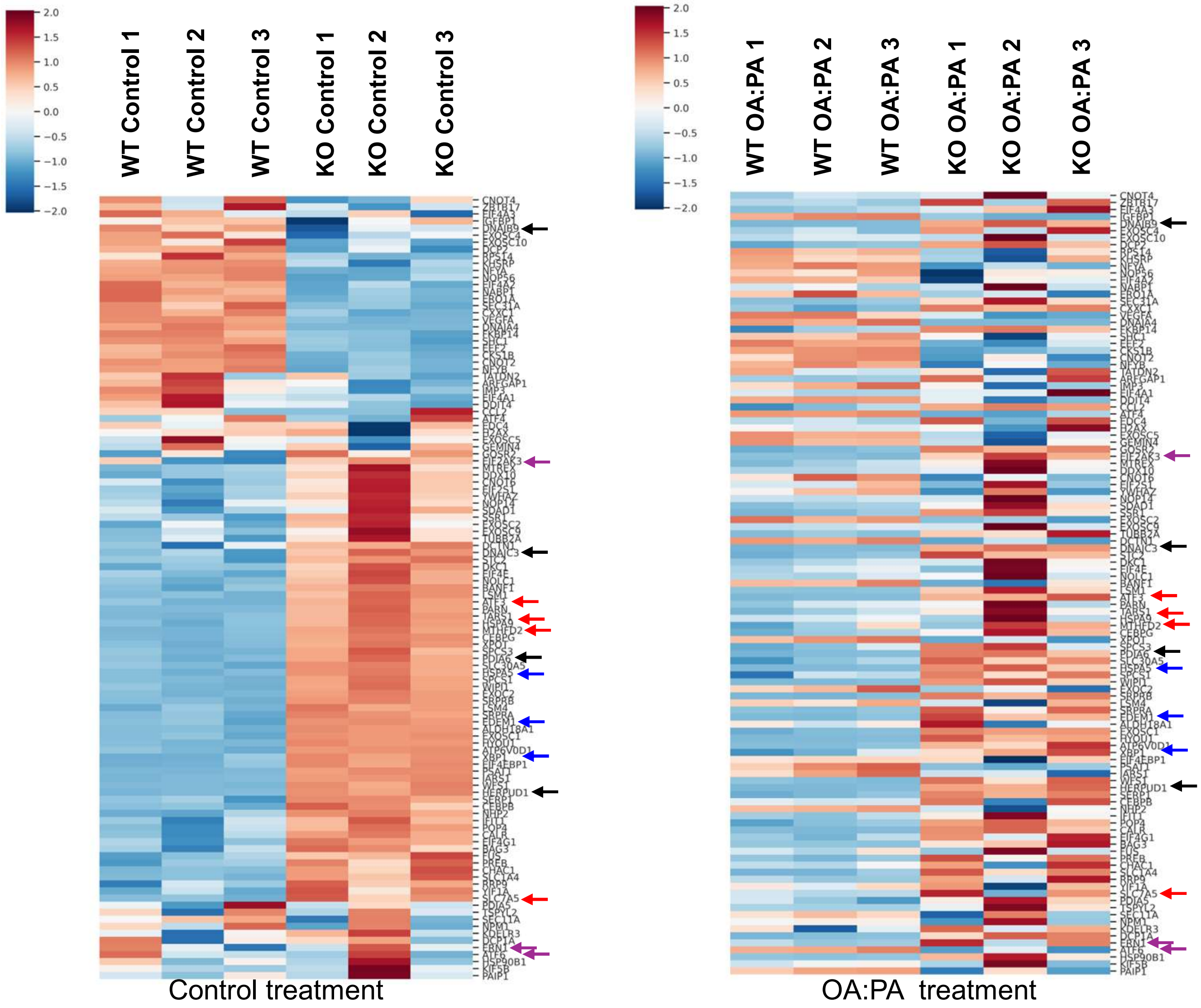

Fig. S5: Peroxisome levels following treatment with OA. wt and PTEN KO HepG2 cells were treated with 0.5 mM OA for 24 h. Representative confocal immunofluorescence images for PEX14 before and after treatment with OA:PA. Quantification of fluorescence intensity shows an increase in PEX14 expression in both wt and KO cells after treatment.

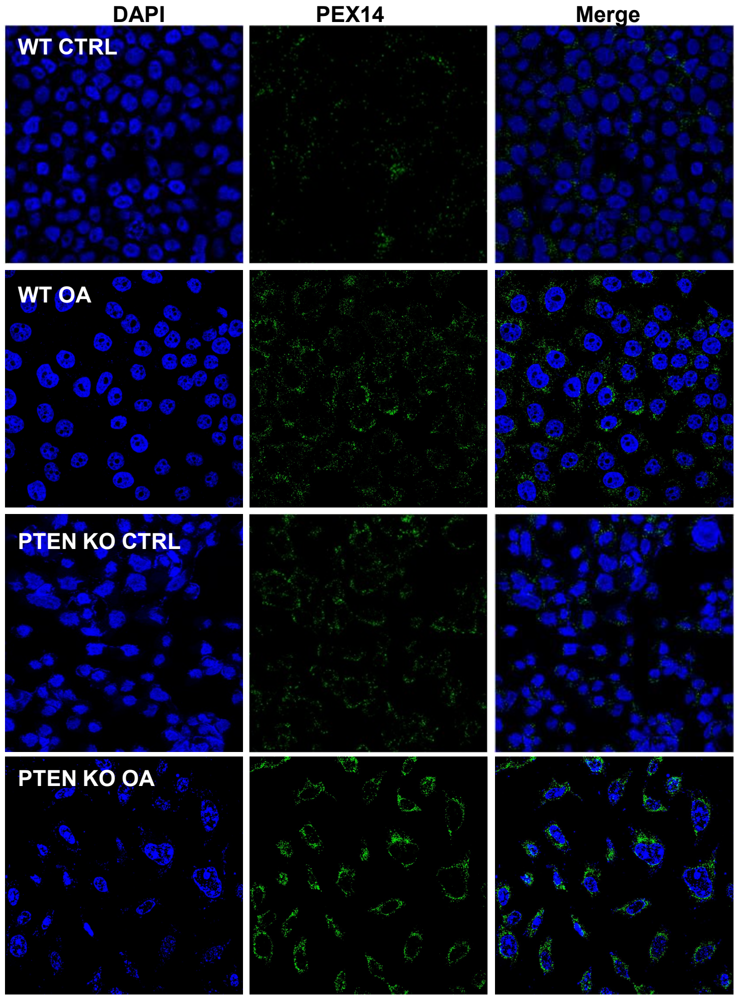

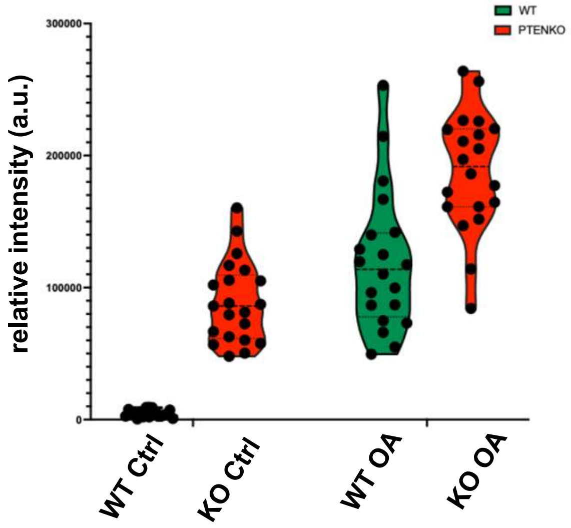

Fig. S6: Differences in the lipid metabolic process between WT and PTEN KO HepG2 following treatment with OA:PA. (A and B) Listed are genes in the triglyceride and **(C and D)** in phosphatidylcholine metabolism pathways clustered by regulation. Relative expression levels before and after treatment with OA:PA is color coded. Genes that are regulated in an opposite manner are labeled by arrows of different colors.

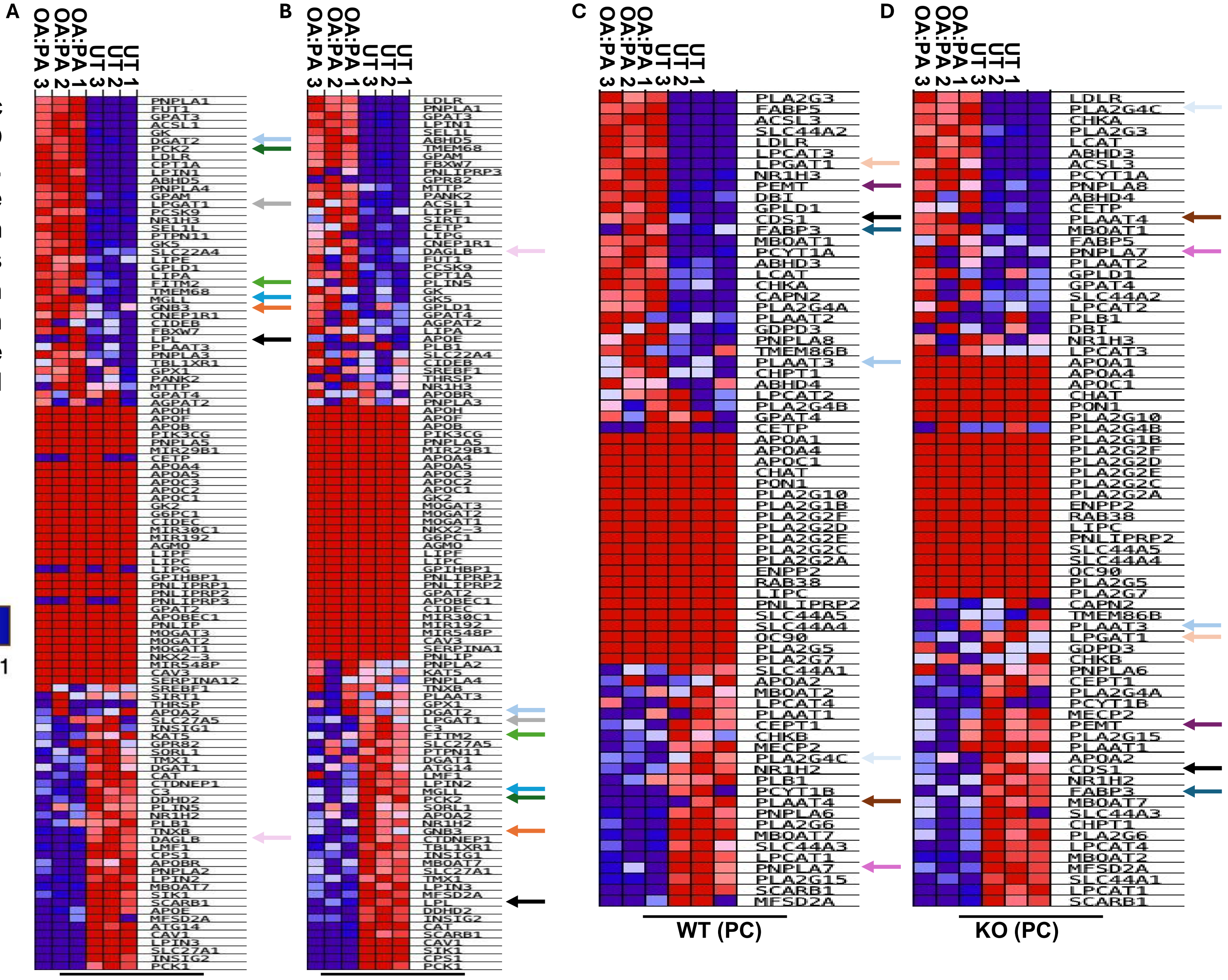

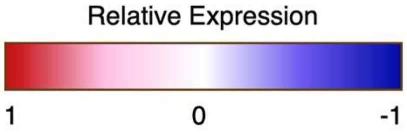

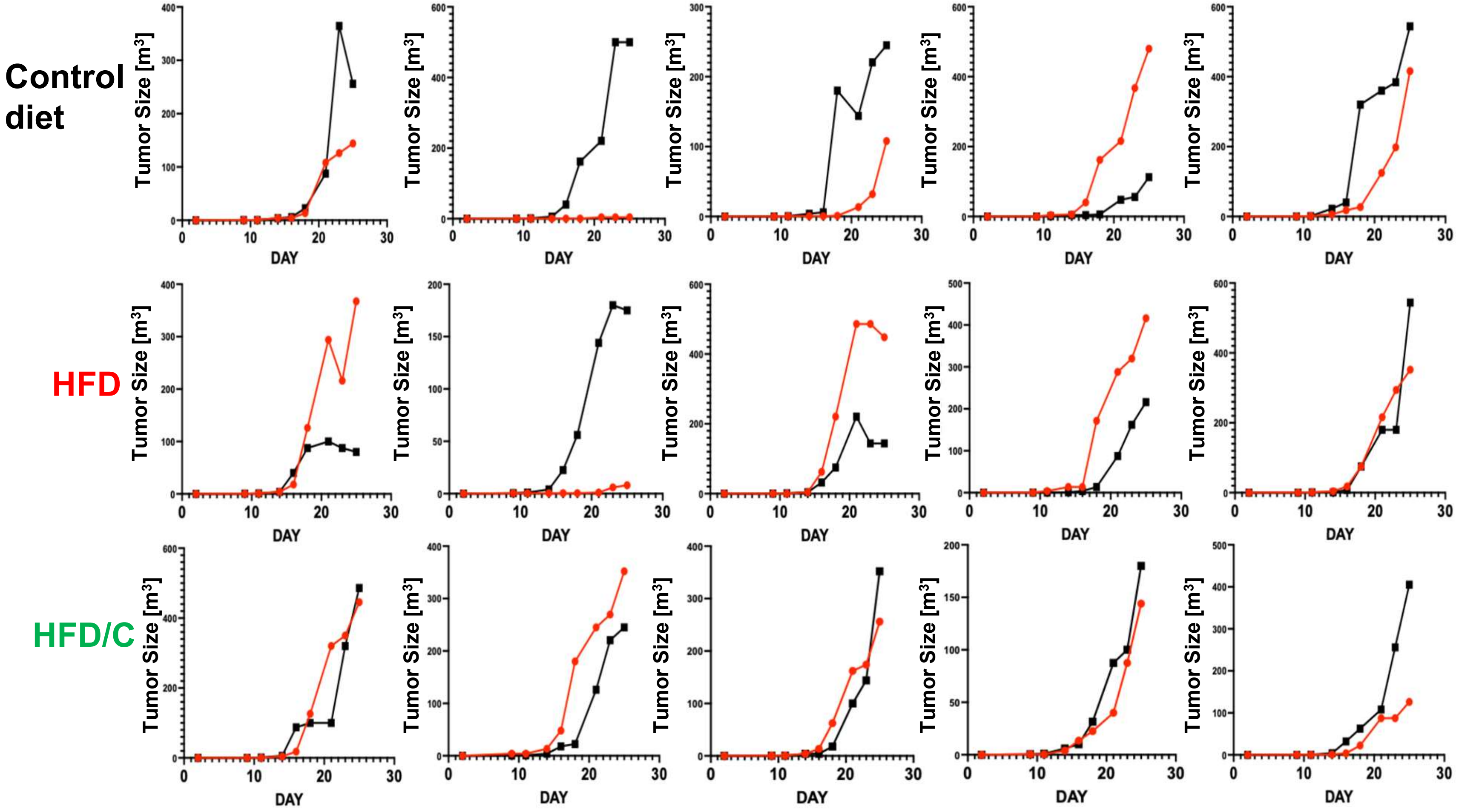

Fig. S7: Xenograft growth in the individual mice. Immunocompromised NSG mice were fed for 4 weeks with control diet, HFD or HFD/C. Mice were challenged in one flank with $1x10^6$ **WT HepG2** cells and $1x10^6$ **PTEN KO HepG2** cells. Tumor growth was monitored and plotted over time for the individual mice. On control diet, in 4/5 mice PTEN KO HepG2 cells grew faster. On HFD, in 3/5 mice **WT HepG2** cells grew faster.
